# Access to means of suicide, occupation and the risk of suicide: a national study over 12 years of coronial data

**DOI:** 10.1186/s12888-017-1288-0

**Published:** 2017-04-04

**Authors:** A Milner, K Witt, H Maheen, AD LaMontagne

**Affiliations:** 1 Centre for Health Equity, Melbourne School of Population and Global Health, University of Melbourne, Melbourne, VIC Australia; 2 Work, Health, & Wellbeing Unit, Centre for Population Health Research, School of Health & Social Development, Deakin University, Melbourne, VIC Australia; 3 Turning Point, Eastern Health Clinical School, Monash University, Melbourne, VIC Australia

**Keywords:** Suicide, Occupation health, Lethal means, Coroners’ data

## Abstract

**Background:**

Availability of lethal means is a significant risk factor for suicide. This study investigated whether occupations with greater access to lethal means had higher suicide rates than those without access, and further, whether this relationship differed for females versus males.

**Methods:**

A retrospective mortality study was conducted across the Australian population over the period 2001 to 2012. Data from the Australian Bureau of Statistics, which collects Census information on occupation for the Australian population, and the National Coroners Information System, which records information on suicide deaths, were combined. Employed suicide records were coded by occupation and work-related access to lethal means. Descriptive analysis and negative binomial regression were used to assess the relationship between access to means and suicide.

**Results:**

Persons in occupations with access to firearms, medicines or drugs, and carbon monoxide more frequently used these methods to end their lives than those without access to means. Females employed in occupations with access to means had suicide rates that were 3.02 times greater (95% CI 2.60 to 3.50, *p* < 0.001) than those employed in occupations without access. Males in occupations with access had suicide rates that were 1.24 times greater than those without access (95% CI 1.16 to 1.33, *p* < 0.001).

**Conclusion:**

Work-related access to means is a risk factor for suicide in the employed population, but is associated with a greater risk for females than males. The findings of this study suggest the importance of controlling access to lethal methods in occupations where these are readily available.

**Electronic supplementary material:**

The online version of this article (doi:10.1186/s12888-017-1288-0) contains supplementary material, which is available to authorized users.

## Background

At a population level reducing access to lethal means is one of few suicide prevention strategies for which there is convincing evidence [1, 2]. Specifically, there have been a number of studies showing that reducing the availability and accessibility of firearms [3], charcoal [4], as well as installing physical barriers and/or safety nets around bridges and tall buildings [5], have been associated with a reduction in suicide deaths, with little evidence of means substitution [5, 6].

The availability of potentially lethal suicide methods has also been cited as an explanation for higher rates of suicide in specific occupational groups [7–12]. Certainly, this may be the case for those working in jobs with ready access to potentially lethal suicide methods, for example, firearms in the case of police and members of the defence force [13], and medicinal drugs in the case of medical and veterinary professionals [8]. However, this explanation is insufficient to explain the elevated suicide rates in other occupations, such as construction workers [14, 15] who would have the same ability to access suicide methods as the general population.

There has been limited research on the relationship between access to means and suicide within the employed population, and those that have been conducted have been based in specific occupational settings (e.g., police, military, doctors and veterinarians) [8, 9, 16]. One of the few studies in this area [17], found that several occupations with access to means actually had lower suicide rates than the employed population. However, this research concluded that access to means played a role in influencing the choice of suicide method within specific occupational groups, rather than being related to an overall greater suicide risk.

The present research seeks to further progress research in this area. At a national level, we seek to assess whether occupations with greater access to potentially lethal suicide methods have elevated suicide rates compared to those that do not have such access. Related to this, we will also explore whether persons employed in occupations with access to specific suicide methods use these methods to end their life. We also seek to assess whether the relationship between occupational access to lethal means and suicide varies for females compared to males. We hypothesise gender as an effect modifier based on past research showing that males and females have different preferences for suicide method, with males tending to use more lethal and violent suicide methods than females [18, 19].

## Methods

### Study design

We conducted a retrospective nationwide study of deaths due to suicide in Australia between 2001 and 2012.

### Ascertainment of suicide deaths

We identified suicide cases using the National Coroners Information System (NCIS). Established in 2001, NCIS captures all reportable deaths in the country, basic demographic information, as well as employment status and occupation at the time of death, collected from coronial files. [20, 21]. The quality and completeness of NCIS data is variable between cases, particularly for the early years of the scheme, and intentional self-harm deaths may be under-reported due to differences in how suicide is determined between coroners and between states [21, 22]. This is because of variation in legislation between state and territories, and differences in this how this applied between coroners. In addition, there is a significant lag-time in reporting of deaths in NCIS due to the lengthy coronial process. As a consequence 2012 was the most recent year available for inclusion in the study. Nevertheless, NCIS offers the best available information on suicide mortality in Australia and is used as the basis for compiling the official death statistics released by the Australia Bureau of Statistics (ABS).

The study included all employed adults with a known occupation who were 15 years of age or older at the time of death. We classified suicide methods according to the International Classification of Disease – 10th revision (ICD-10) within method specific codes X60-X84 [23].

### Ascertainment of occupational group

Occupational information was coded according to the Australian and New Zealand Standard Classification of Occupations (ANZSCO) to the four-digit level [24]. Information on the coding procedure utilised for this study can be found in Additional file 1.

### Occupations that have lethal access to means

Occupations with access to lethal means (OAM) represented those that: 1) had greater access to lethal means in the course of their work, and; 2) had knowledge about how to use these lethal means to harm themselves. We particularly considered the following suicide methods: firearms, medications and other drugs, carbon monoxide, as well as access to poisons. These methods, and the occupations classified as having access to these, can be seen in Table 1.

**Table 1:** Occupations classified as having access to lethal means of suicide

Suicide methods	Occupation (with ANZSCO code)
Firearms	Farmers
	Farmers and Farm managers (ANZSCO l)
	Protective Service Workers (ANZSCO 44)
	Livestock Farm Workers (ANZSCO 8415)
	Mixed Crop & Livestock Farm Workers (ANZSCO 8416)
	Other Farm, Forestry & Garden Workers (ANZSCO 8419)
	Defence Force Members (ANZSCO 441 l)
	Fire and Emergency Workers (ANZSCO 4412)
	Police (ANZSCO 4413)
	Prison Officers (ANZSCO 4421)
	Security Officers and Guards (ANZSCO 4422)
Medicine and drugs	Veterinarians (ANZSCO 2347)
	Pharmacists (ANZSCO 2515)
	Dental Practitioners (ANZSCO 2523)
	Medical Practitioners (ANZSCO 253)
	Midwifery and Nursing Professionals (ANZSCO 254)
	Chemists (ANZSCO 2342 and 3114)
Poisons (cleaning products and pesticides)	Industrial cleaners (ANZSCO 8112 or 8113).
	Farm, Forestry and Garden Workers
	Farmers and Farm managers (ANZSCO l)
	Livestock Farm Workers (ANZSCO 8415)
	Mixed Crop & Livestock Farm Workers (ANZSCO 8416)
	Other Farm, Forestry & Garden Workers (ANZSCO 8419)
	Crop Farm Workers (ANZSCO 8412)
	Forestry and Logging Workers (ANZSCO 8413)
	Garden and Nursery Labourers (ANZSCO 8414)
Carbon Monoxide	Automobile Drivers (ANZSCO 7311)
	Bus and Coach Drivers (ANZSCO 7312)
	Panelbeaters, & Automotive/Engineering Trades (ANZSCO 32)
	Delivery Drivers (ANZSCO 732)
	Truck Drivers (ANZSCO 733)
	Vehicle Body Builders, Trimmers & Painters (ANZSCO 324)

*Notes:* Other Farm, Forestry & Garden Workers (ANZSCO 8419) also includes Pest Controller, and Farm, Forestry and Garden Workers not elsewhere classified

### Population level data

Population estimates for calculating rates in different ANZSCO occupational groupings were obtained from the ABS using the 2006 census data (the mid-point of the study) [25].

### Analysis

#### Descriptive analysis and age-standardised suicide rates

We descriptively assessed whether OAM used specific lethal means to end their lives. Male and female suicide rates per 100,000 persons were calculated for OAM and occupations with no access to means (ONAM). Rates were age-standardised using the Australian standard population as at 2001 [26].

#### Regression models

Negative binomial regression analysis was used to compare whether those OAM had higher rates of suicide than ONAM. We chose negative binomial regression after assessing the extent of over-dispersion in Poisson regression models. We then examined bivariate relationships between the following possible covariates: age, sex, occupational skill level (ANZSCO groups 1 to 8), and year of death. Following this, we assessed whether OAM had higher rates of suicide than ONAM, while controlling for occupational skill level, age and gender. Effect modification by gender was tested by including an interaction term with access of means in the model. For all models, coefficients were transformed into Incidence Rate Ratios (IRRs) to aid interpretation. Analysis was undertaken in Stata for Mac, version 13.1 [27].

## Results

As can be seen in Table 2, over 30% of males in OAM with access to firearms and medicine and drugs used these methods to end their lives, while over 20% used carbon monoxide poisoning as compared to 7.2, 4.5, and 16.9% respectively of ONAM. However, hanging was the most prevalent method for the majority of suicides.

**Table 2:** Proportion of suicides that used work-related means to suicide, within categories classified as having and not having access to these lethal means of suicide, 2001 to 2012, males, Australia

	Occupations without access	Occupations with access to firearms	Occupations with access to medicine and drugs	Occupations with access to carbon monoxide	Occupations with access to poisons
Males (*n* = 8600)	*n* = 6475	*n* = 576	*n* = 153	*n* = 1084	*n* = 312
Firearms	7.17	30.38	6.54	7.56	13.14
Medicine and drugs	4.51	3.30	35.29	4.06	6.09
Carbon monoxide poisoning	16.86	12.85	9.15	20.30	18.59
Poisons	5.08	2.08	2.61	4.24	1.60
Hanging	50.32	38.72	27.45	51.11	51.28
Driving/hit by vehicle	2.50	1.39	4.58	2.03	1.92
Jumping	3.92	0.87	6.54	1.75	1.92
Drowning	1.16	0.69	0.65	1.11	0.64
Other	8.48	9.72	7.19	7.84	4.81

*Notes:* “Other” methods includes a small number of suicide methods such as cutting, death by immolation, death by electrocution, and missing methods

Table 3 indicates that around one-half (46%) of females employed in OAM used medications and 50% of OAM with access to carbon monoxide (albeit based on only 12 cases) used these methods to end their lives, compared to 18 and 14.7% of those employed in ONAM. A greater proportion of females in OAM used firearms (17.4%) compared to 2.4% of ONAM.

**Table 3:** Proportion of suicides that used work-related means to suicide, within categories classified as having and not having access to these lethal means of suicide, 2001 to 2012, females, Australia

	Occupations without access	Occupations with access to firearms	Occupations with access to medicine and drugs	Occupations with access to carbon monoxide	Occupations with access to poisons
Females (*n* = 1550)	*n*=1236	*n* = 46	*n* = 192	*n* = 12	*n* = 64
Firearms	2.43	17.39	0.52	0.00	3.13
Medicine and drugs	17.96	8.70	46.35	16.67	17.19
Carbon monoxide poisoning	14.72	8.70	10.42	50.00	14.06
Poisons	1.05	4.35	0.52	0.00	1.56
Hanging	43.04	41.30	27.60	16.67	45.31
Driving/hit by vehicle	2.51	2.17	1.56	0.00	6.25
Jumping	7.77	2.17	2.08	0.00	6.25
Drowning	3.24	4.35	4.17	8.33	3.13
Other	7.28	10.87	6.77	8.33	3.13

*Notes:* “Other” methods includes a small number of suicide methods such as cutting, death by immolation, death by electrocution, and missing methods

Table 4 describes age-standardised rates of suicide per 100,000 for OAM and ONAM by gender. As can be seen, those persons working in OAM had higher rates of suicide (both males and females). However, the magnitude of these differences varied by gender. Females employed in OAM had about twice the suicide rate of those employed in ONAM. Males employed in OAM, on the other hand, had only slightly elevated rates compared to ONAM.

**Table 4:** Suicide rates within occupations with and without access to lethal means, by gender, 2001 to 2012

	ASR	Lower CI	Upper CI
Males, no access	11.6	11.3	11.9
Males, access	15.7	14.9	16.4
Females, no access	3	2.6	3.4
Females, access	7.6	6.4	8.9

*Notes: ASR* age-standardised suicide rates expressed as per 100,000 person years. *Lower CI* 95% CI Interval at the lower bound; *Upper CI* 95% CI Interval at the upper bound

We next conducted negative binomial regression testing whether the relationship between access to means and suicide varied by gender. The interaction term was significant (females in OAM had suicide rates 2.15 higher than males in ONAM, 95% CI 1.84 to 2.52, *p* < 0.001). The model with the interaction term was also provided significantly better fit compared to a model without the interaction term (LR χ^2^ = 84.60, *p* < 0.001). On the basis of this, we stratified analyses by gender (Table 5). Table 5 shows that access to means was associated with a 3.02 RR of suicide for females (95% CI 2.60, 3.50, *p* < 0.001) and a 1.24 RR of suicide for males (95% CI 1.16, 1.33, *p* < 0.001), after controlling for year of death, occupational skill level and age (only the adjusted models are presented here for the sake of brevity). We would note that the male RR associated with some categories of occupational skill level was substantially higher than the RR associated with OAM.

**Table 5:** Negative binominal regression model, suicide within occupations with and without access to lethal means, controlling for potential confounders, by gender, 2001 to 2012

	Males					Females				
	Suicide	Pop (‘000)	IRR	95% CI	*p* value	Suicide	Pop (‘000)	IRR	95% CI	*p* value
Occupations with access to lethal means										
Yes	2125	912	1.24	1.16, 1.33	<0.001	314	426	3.02	2.60, 3.50	<0.001
No	6475	3900	1			1236	3700			
Age group										
70 yrs. +	81	52	1.90	1.48, 2.43	<0.001	4	23	5.49	2.02, 14.87	0.001
60–69 yrs	450	328	0.81	0.72, 0.92	0.001	61	193	1.71	1.30, 2.26	<0.001
50–59 yrs	1406	917	0.83	0.76, 0.91	<0.001	287	776	0.92	0.79, 1.08	0.326
40–49 yrs	2344	1146	1.08	0.99, 1.18	0.066	406	1042	0.89	0.77, 1.03	0.106
30–39 yrs	2303	1127	1.07	0.98, 1.17	0.118	442	930	1.09	0.94, 1.25	0.243
10–29 yrs	2016	958	1			350	869	1		
Occ skill level										
Labourers	1758	605	2.37	2.12, 2.64	<0.001	153	348	1.13	0.89, 1.43	0.319
Machine workers	1111	542	1.47	1.31, 1.64	<0.001	23	63	3.13	2.01, 4.88	<0.001
Sales workers	418	343	1.13	0.97, 1.30	0.107	153	553	1.09	0.86, 1.38	0.464
Clerical and Admin	331	317	0.82	0.70, 0.96	0.012	287	1049	0.81	0.65, 0.99	0.041
Community service	612	250	1.88	1.65, 2.13	<0.001	305	552	1.70	1.39, 2.09	<0.001
Technical and trade	2412	1116	1.59	1.43, 1.77	<0.001	94	193	1.80	1.38, 2.35	<0.001
Professionals	940	850	0.87	0.77, 0.98	0.026	401	956	0.80	0.66, 0.98	0.033
Managers	1018	789	1			134	414	1		
Year	8600	4812	0.97	0.96, 0.98	<0.001	1550	4126	0.98	0.96, 0.99	<0.001

*Notes: 95% CI Intervals* 95% Confidence intervals (lower, upper), *IRR* incident rate ratio (suicides per 100,000 person-years), *p value* significance value at 95%; Occupations with access to lethal means can be seen in Table 1

## Discussion

Our results suggest that occupational access to means is an important risk factor for suicide. Those occupations characterized by greater access and familiarity with potentially lethal suicide methods had overall higher suicide rates than those without. Females with access were at particularly elevated risk compared to men. At the same time, we would acknowledge that, within the employed population, males and females tend to use similar suicide methods, with hanging being the most common method choice for most groups, followed by carbon monoxide poisoning.

In stating these results, our paper has a number of limitations that need to be taken into consideration. First, the underreporting of suicide (because of misclassification of cause of death) can be expected in NCIS, as in any official record of death. Second, occupation may have been misreported by police collecting information on occupation or in the coding process, which may have occurred despite independent coding by two researchers and the use of a structured approach to classification. Third, data on suicide methods is incomplete as initial data collection from NCIS did not include information on suicide methods. We have subsequently obtained this data for most cases but were unable to match suicide method for a small proportion of cases (approximately 10%). It is also worth note that NCIS is not an open data source. Fourth, there are several confounders that we were unable to control for due to restrictions in the data set and lack of exposure information (e.g., psychosocial job stressors, other stressors outside the workplace). Last, we have taken an overly inclusive approach to the classification of OAM. Thus we believe, if anything, we have over-represented occupations with access and knowledge about suicide methods. This would have the effect of biasing our results towards the null. We would also note that the descriptive analysis reported in Table 2 was based on a small number of cases for females, and thus we would caution against over-interpretation of results (e.g., occupations with access to carbon monoxide poisons being based on 12 cases only). The major strengths of our study include the use of the best available individual-level data on suicide and coverage across an entire national population over a twelve-year period.

Factors influencing an individual’s choice of suicide method are believed to include availability, knowledge about how to use a potential suicide method, and the overall perceived cultural acceptability of the method [28–31]. For example, there was a substantial reduction in firearm suicides in Australia after implementation of national policy restricting access to guns [32]. At the same time, there has been an increase in hanging, which is possibly because this method is more difficult to regulate [32]. It is also possible that stigma about hanging and criminal behaviour have reduced followed the cessation of capital punishment [33]. These issues are important from a prevention perspective, considering hanging is the most commonly used method in Australia. A previous study conducted with a group of suicide survivors who used carbon monoxide poisoning indicated several other risk factors, including awareness of the method through media sources [34]. This aligns with other research which has shown that media portrayal of certain methods is an important factor in the decision to use that specific method [35]. It is important to note that most past research about choice of suicide means has been conducted at the general population level or within specific clinical samples. There has been comparatively limited research on the use of specific methods within occupational groups. Despite this, we would argue that similar explanations (e.g., ready access to potentially lethal suicide methods and greater knowledge about how to use these) are likely to comprise part of the explanation for our results. However, factors influencing choice of one method over another may be particularly pertinent as a risk within occupational settings compared to the general population, given that workers in OAM may use these potential suicide methods in their everyday work. For example, doctors have substantial knowledge about the toxicity of prescription medications and the amount that would be required to result in serious harm or death.

Choice of suicide method is also likely reflects a complex number of gender-specific factors. Previous research suggests that a preference for a quick and painless death and the use of method that entails no risk of disfigurement have been found to be important influences on method choice for females [28]. Our results suggest that, where they have ready access, employed females will use methods such as carbon monoxide poisoning and drug overdose rather than hanging. They may have chosen these methods because, as suggested above, they are associated with less pain and disfigurement than other methods. In saying this, a greater proportion of females who had occupational access to firearms used these to end their lives, despite the fact that this method is potentially associated with considerable disfigurement. This latter finding, and the fact there is a limited amount of research on gender differences in method choice within the employed population, highlights the need for future research on this topic.

OAM have been identified as being “at risk” in several previous studies [8, 9, 16, 17, 36]. The reasons for this have been linked to a variety of factors, including adverse working contexts, selection issues into the occupation, as well as access to lethal means. We would highlight the fact that we found elevated suicide rates among several occupational groups without access to lethal means, such as labourers (males) and machinery worker (females), aligning with our past research [37, 38]. We controlled for occupational skill level in the present analysis to account for potential confounding by socio-economic position as well as by other work-related factors that pattern by occupational status in Australia and internationally, such as exposure to poor psychosocial working conditions, or job stressors [39]. We acknowledge there is undoubtedly a number of complex factors underpinning suicide in the employed population aside from access to lethal means. Our results, nevertheless, suggest that means access appears to play a role for some occupational groups more than others.

## Conclusion

The findings of this study suggest the importance of controlling access to lethal methods in occupations where these are readily available, and where there is evidence that these are particularly utilised by those who die by suicide. These prevention initiatives need to be combined with strategies that address other risk factors within these occupations, including adverse working conditions [40, 41]. Risk factors are likely to vary between different occupations. This highlights the importance of prevention initiatives being tailored for specific occupational groups. Our results also indicate that even within a specific OAM, there may be a need for different interventions for males and females.

## Additional file

Additional file 1: Information on the coding of occupation.” Provides additional data on how the suicide data was coded by occupation. (DOCX 15 kb)

## Abbreviations

ABS: Australia Bureau of Statistics
ANZSCO: The Australian and New Zealand Standard Classification of Occupations
ICD-10: International Classification of Disease – 10th revision
NCIS: National Coroners Information System
OAM: Occupations with access to lethal means
ONAM: Occupations with no access to means
RR: Rate-ratio
